# Developing standards for the microbiome field

**DOI:** 10.1186/s40168-020-00856-3

**Published:** 2020-06-26

**Authors:** Gregory C. A. Amos, Alastair Logan, Saba Anwar, Martin Fritzsche, Ryan Mate, Thomas Bleazard, Sjoerd Rijpkema

**Affiliations:** 1 Division of Bacteriology, National Institute for Biological Standards and Control, Blanche Lane, South Mimms, Potters Bar, Hertfordshire, EN6 3QG UK; 2 Division of Analytical and Biological Sciences, National Institute for Biological Standards and Control, Potters Bar, Hertfordshire, EN6 3QG UK

## Abstract

**Background:**

Effective standardisation of methodologies to analyse the microbiome is essential to the entire microbiome community. Despite the microbiome field being established for over a decade, there are no accredited or certified reference materials available to the wider community. In this study, we describe the development of the first reference reagents produced by the National Institute for Biological Standards and Control (NIBSC) for microbiome analysis by next-generation sequencing. These can act as global working standards and will be evaluated as candidate World Health Organization International Reference Reagents.

**Results:**

We developed the NIBSC DNA reference reagents Gut-Mix-RR and Gut-HiLo-RR and a four-measure framework for evaluation of bioinformatics tool and pipeline bias. Using these reagents and reporting system, we performed an independent evaluation of a variety of bioinformatics tools by analysing shotgun sequencing and 16S rRNA sequencing data generated from the Gut-Mix-RR and Gut-HiLo-RR. We demonstrate that key measures of microbiome health, such as diversity estimates, are largely inflated by the majority of bioinformatics tools. Across all tested tools, biases were present, with a clear trade-off occurring between sensitivity and the relative abundance of false positives in the final dataset. Using commercially available mock communities, we investigated how the composition of reference reagents may impact benchmarking studies. Reporting measures consistently changed when the same bioinformatics tools were used on different community compositions. This was influenced by both community complexity and taxonomy of species present. Both NIBSC reference reagents, which consisted of gut commensal species, proved to be the most challenging for the majority of bioinformatics tools tested. Going forward, we recommend the field uses site-specific reagents of a high complexity to ensure pipeline benchmarking is fit for purpose.

**Conclusions:**

If a consensus of acceptable levels of error can be agreed on, widespread adoption of these reference reagents will standardise downstream gut microbiome analyses. We propose to do this through a large open-invite collaborative study for multiple laboratories in 2020.

Video Abstract

## Background

Developments in next-generation sequencing (NGS) technologies have facilitated the rapid expansion of the microbiome field. As new technologies have been developed, the cost per read of sequencing has decreased, meaning sequencing-based cohort studies have become more accessible to the wider scientific community [1]. NGS technologies have allowed for microbiome studies from vast cohorts of volunteers and patients [2, 3]; however, differences across methodologies have led to uncertainty on best practice approaches for studying the microbiome [4]. For example, studies have established significant variation in results occur when using different microbiome protocols to analyse the same samples [5–7]. This has been attributed to multiple reasons including differences in storage and collection of the sample [8], differences across different DNA extraction procedures [6], differences across the available NGS platforms commonly used for sequencing [9], differences between using a shotgun sequencing approach vs an amplicon approach [7], differences between the different 16S rRNA regions which can be amplified when a 16S rRNA approach is being used [10], and differences in bioinformatics pipelines depending on both laboratory preference and the current prevailing consensus can also introduce significant biases into results [5].

Despite efforts to better standardise the microbiome field, there are no certified or accredited reference reagents available and no framework established for how potential reference reagents could work. Without reference reagents, standardisation of the microbiome field remains impossible, as users are not able to accurately evaluate their pipelines or use established controls for their experiments. Reference reagents which are widely used by the field are often termed ‘standards’. In many fields, standards are the bedrock of clinical trials and product manufacture, allowing commutability between results from globally disparate groups conducting clinical trials and giving assurances of quality during product manufacture [11]. For users, standards allow for a critical evaluation of the procedures being used, a better understanding of where biases have been introduced, and if applicable, calibration of an assay to allow results to be interpreted in a standard arbitrary unitage [11]. The creation of global standards for the microbiome field has the potential to improve method development, prevent erroneous results being reported, and allow for effective commutability of results globally. These improvements will be essential for effective translation of research to clinical application. Furthermore, standards can open up innovation in the field, as they negate the requirement for everyone to use the same protocol as long as users validate their protocol with respect to the global standard [11].

Standardisation of the microbiome field is complex: methodologies used for study comprise multiple steps and the primary measurements are semi-quantitative estimates of thousands of a priori unknown components. Hence, for effective standardisation, there likely needs to be a range of reference reagents to standardise the multiple steps which introduce variability into studies [4]. Furthermore, there needs to be a robust reporting framework which allows measurement and evaluation of how accurately methods are capturing the abundance of known and a priori unknown microbes. The National Institute for Biological Standards and Control (NIBSC) is a collaborating centre of the World Health Organization (WHO) and produces and stocks the majority of WHO International Reference Reagents or international standards. WHO standards are widely considered as references of the highest order and these allow the assessment of the commutability of studies on a global scale [12]. As part of NIBSC’s role in the development of WHO reference reagents, we have initiated a substantial program to create a suite of microbiome reference reagents and a reporting framework for effective standardisation of the field. Here, we set out the creation of the first NIBSC DNA reference reagents for analysis of the microbiome and detail a robust reporting framework for the evaluation of biases in common analyses. These DNA reference reagents are to be used as global working standards for gut microbiome analysis by NGS and are considered as future candidates for International Reference Reagents, which will be evaluated in multiple laboratories and put forward for endorsement by the WHO. Using our reference reagents and a reporting system developed in-house, we performed several benchmarking studies of common 16S rRNA and shotgun pipelines. Currently, there are a few reference reagents commercially available which are marketed for microbiome standardisation. We also evaluated these reference reagents for benchmarking pipelines and demonstrate how reagent composition of the reference impacts the results of benchmarking studies.

## Results

### Development of reference reagents and a reporting system

It is envisaged that at least three types of reference reagent will be required for effective standardisation of microbiome protocols: DNA reagents to control for biases in library preparation, sequencing, and bioinformatics pipelines; whole-cell reagents to control for biases in DNA extraction; and matrix-spiked whole-cell reagents to control for biases from inhibitors or storage conditions. In this study, we created DNA reference reagents which allow for the standardisation of downstream analyses.

Establishing reference reagents for the analysis of the microbiome requires the construction of reagents of known composition (‘ground truth’) which can be used to evaluate the accuracy of the predicted taxonomic composition given by pipelines used for microbiome analysis. We created two DNA mock communities, Gut-Mix-RR and Gut-HiLo-RR, consisting of 20 common gut microbiome strains in both an even and staggered composition (Table 1). These reference reagents comprised strains spanning 5 phyla, 13 families, 16 genera, and 19 species, to allow testing of pipelines’ ability at different taxonomic levels (Table 1) [13]. A key component of a reference reagent is to have a reproducible reporting system allowing a global comparison of results. Because no reporting system currently exists, an in-house reporting system was designed to assess downstream microbiome analyses.

**Table 1:** Strains and characteristics of the NIBSC Gut-Mix-RR and Gut-HiLo-RR. Gut-Mix-RR percentage is based on relative number of genome copies. Gut-HiLo-RR percentage is based on relative number of genome copies. GC content based on genome sequences where available or if not available (*) by the original species description. Accession numbers are GenBank Accession numbers or RefSeq accession numbers if available. NC_008530 is the RefSeq accession for the type strain *Lactobacillus gasseri* ATCC 33323*.* Sequences generated from this study for all strains are available from NCBI Bioproject ID PRJNA622674. 16S rRNA copy number and intragenomic variation is based on analysis of genome sequences through IMG/M

Species	Culture collection number	GC-content (%)	Gut-Mix-RR (%)	Gut-HiLo-RR (%)	Accession numbers	16S copy number (number of sequence variants)
*Akkermansia muciniphila*	DSM 22959	55.8	6.37	0.18	NC_010655	3 (1)
*Alistipes finegoldii*	DSM 17242	56.7	4.54	1.30	NC_018011	2 (2)
*Anaerostipes hadrus*	DSM 3319	37.2	6.11	1.75	NZ_KB290627	1 (1)
*Bacteroides thetaiotaomicron*	DSM 2079	42.9	2.69	7.72	NC_004663	5 (3)
*Bacteroides uniformis*	DSM 6597	46.5	3.66	1.05	GCF_000154205	4 (2)
*Bifidobacterium longum subsp. infantis*	DSM 20088	59.9	6.00	17.20	NC_011593	4 (1)
*Bifidobacterium longum subsp. longum*	DSM 20219	60.3	6.92	19.82	GCF_900104835	4 (2)
*Blautia wexlerae*	DSM 19850	41.4	3.77	0.11	GCF_000484655	2 (1)
*Clostridium butyricum*	DSM 10702	28.5	3.70	10.59	GCF_000409755	1 (1)
*Collinsella aerofaciens*	DSM 13712	60.0*	6.95	1.99	GCF_902501475	7 (3)
*Escherichia coli*	DSM 1103	50.4	3.26	9.33	CP009072	7 (5)
*Eubacterium hallii*	DSM 3353	38.2	5.16	1.48	GCF_000173975	1 (1)
*Faecalibacterium prausnitzii*	DSM 17677	56.4	5.49	0.16	NZ_CP048437	3 (3)
*Lactobacillus gasseri*	DSM 20077	33.0	8.97	0.26	NC_008530	6 (6)
*Parabacteroides distasonis*	DSM 20701	45.1	3.52	10.10	NC_009615	7 (3)
*Prevotella copri*	DSM 18205	44.9	4.83	13.84	GCF_000157935	7 (6)
*Prevotella melaninogenica*	DSM 7089	41.0	5.34	1.53	NC_014370.1 and NC_014371.1	4 (2)
*Roseburia hominis*	DSM 16839	48.5	4.72	1.35	NC_015977	4 (2)
*Roseburia intestinalis*	DSM 14610	42.6	3.88	0.11	NZ_LR027880	6 (5)
*Ruminococcus gauvreauii*	DSM 19829	47.6	4.13	0.12	GCF_000425525	4 (3)

When developing our reporting system, we considered measures which could capture biases that are introduced commonly during analytical pipelines and reflect the results that are reported during microbiome studies. It is important to note the different requirements for developing a reporting framework to allow for effective standardisation in contrast to measures for evaluating taxonomic classifier performance. When assessing classifier performance, area under the precision-recall curve (AUPR) has been successfully used as a measure of how well classifiers detect strains and introduce false positives at different abundance thresholds^21^. However, for standardisation, it is important to delineate these two separate biases to allow adequate control over the separate aspects of the sensitivity in detecting species present and the introduction of false positives into the dataset. To ensure methodologies are fit for purpose, our reporting framework also needed to capture the accuracy of pipelines in reporting species composition and species diversity, as these are measures reported by many microbiome studies. The resulting reporting framework for use with the NIBSC reference reagents consisted of four key measures. To measure how well microbiome analytical pipelines detect known species in a sample, we chose the measure of the number of correctly identified species in the reagent expressed as a percentage. This is often termed true positive rate, recall, or *sensitivity*. We chose the term sensitivity for reporting purposes as it describes the sensitivity in detecting strains. False-positive species can be either measured by the actual number of false positives detected or as the total relative abundance of false positives in the final species composition. For microbiome researchers, a single false-positive species which is of high abundance in the final dataset is arguably more problematic than two false-positive species of a very low abundance. Therefore, we chose the total relative abundance of false-positive species to assess how analytical pipelines introduced false-positive species and termed this false positive relative abundance (*FPRA*) for reporting purposes. Commonly, microbiome studies report on species diversity as a key metric for health. For this reason, we wanted a measure of how well analytical pipelines determined the alpha diversity in a sample. Studies can often use a wide range of alpha-diversity metrics depending on the purpose of the study. We chose the measure of the observed number of total species as it gives users of the reagents a direct measure of whether they are underestimating or overestimating the number of species present in their sample. This reporting measure is hereon termed *diversity.* Finally, we wanted to assess how well pipelines predicted species composition reflects the actual species composition of a sample. To measure this, we used the Bray-Curtis similarity index and termed it *similarity* for reporting purposes (Supplementary Material). The Bray-Curtis similarity index was chosen as it is one of the most highly cited methods for evaluating species composition [14]. We note that there a number of other measures which could be useful in assessing pipeline accuracy, and these could be added to these four measures by users if desired.

### Evaluation of shotgun sequencing for taxonomic profiling using NIBSC RRs

**Fig. 1:**
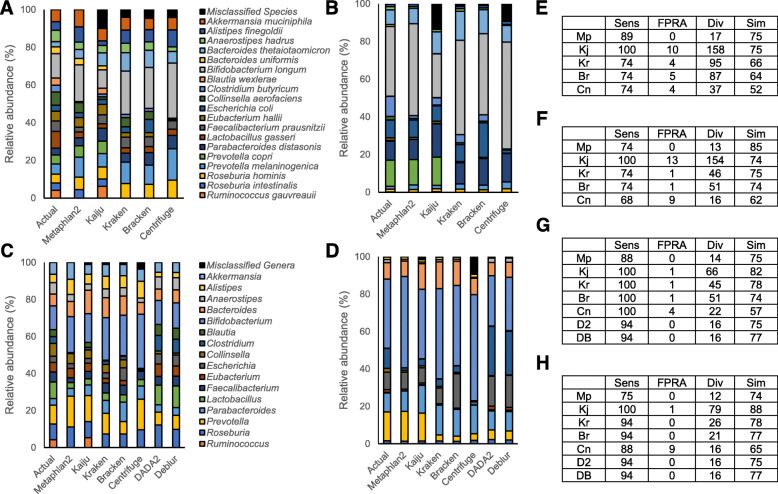
A comparison of different bioinformatics tool performances at both the species level and genera level using the NIBSC Gut-Mix-RR and Gut-HiLo-RRs. **a** Relative abundance of each species in the Gut-Mix-RR as calculated by five different metagenomic taxonomic profiling tools in comparison to the known composition of the reagent. **b** Relative abundance of each species in the Gut-HiLo-RR as calculated by five different metagenomic taxonomic profiling tools in comparison to the known composition of the reagent. **c** Relative abundance of each genera in the Gut-Mix-RR as calculated by five different metagenomic taxonomic profiling tools and two 16S rRNA taxonomic profiling pipelines in comparison to the known composition of the reagent. **d** Relative abundance of each genera in the Gut-HiLo-RR as calculated by five different metagenomic taxonomic profiling tools and two 16S rRNA taxonomic profiling pipelines in comparison to the known composition of the reagent. **e** Reporting measures for pipeline performance for calculating species as evaluated using the Gut-Mix-RR. **f** Reporting measures for pipeline performance for calculating species as evaluated using the Gut-HiLo-RR. **g** Reporting measures for pipeline performance for calculating genera as evaluated using the Gut-Mix-RR. **h** Reporting measures for pipeline performance for calculating genera as evaluated using the Gut-HiLo-RR. Mp MetaPhlAn2, Kj Kaiju, Kr Kraken, Br Bracken, Cn Centrifuge, Sens sensitivity, FPRA false positive relative abundance, Div diversity, Sim similarity

Few studies have independently compared the bioinformatics tools used for taxonomic profiling of shotgun sequencing data. To validate the suitability of the NIBSC-Gut-Mix-RR and NIBSC-Gut-HiLo-RRs and reporting system, we conducted a study into the variability in outputs across bioinformatics tools used to profile the taxonomy of shotgun sequencing metagenomic datasets. Shallow shotgun sequencing [15] was performed on five replicates of the reference reagents. Following initial quality control, sequencing data was subsampled to 500,000 reads per replicate and analysed using five common bioinformatics tools which profile taxonomy from metagenomes, MetaPhlAn2 [16], Kraken [17], Bracken [18], Kaiju [19], and Centrifuge [20] (Fig. 1, Supplementary Table 1). Bioinformatics tools were tested following the developer’s recommended settings to ensure a fair comparison between methods, and examples of commands ran can be found in Supplementary Methods. Across all tested tools and reagents, there was very little variation across biological replicates (Supplementary Table 1). At the species level, taxonomic profilers significantly differed across all reporting measures for both reference reagents (Fig. 1, Supplementary Table 2). There was a notable trade-off between sensitivity and FPRA, with the most sensitive tool Kaiju having the highest FPRA, and the tool with the lowest FPRA MetaPhlan2 having reduced levels of sensitivity. For the GutMix-RR, sensitivity ranged from 73 to 100% across the five bioinformatics tools with Kaiju being the only bioinformatics tool to detect all species in the reagent. This was primarily due to problems detecting *Blautia wexlerae* and *Ruminococcus gauverauii*, with some tools misclassifying them as incorrect species in the correct genera (e.g. Kraken, Bracken, Centrifuge) and others failing to detect any species in the entire genera (MetaPhlAn2). MetaPhlAn2 was the only bioinformatics tool with 0% FPRA, with multiple tools detecting a high number of incorrect species at low abundances. For example, Kraken and the related Bracken both detected 13 different Bacteroides species, when only two were present. Centrifuge incorrectly assigned a large proportion of *Escherichia* to the genus *Shigella*, whilst Kaiju detected many species, unrelated to genera in the reference reagent, at low abundances. Differences in sensitivity and FPRA across the tools led to differences in diversity and similarity. In particular, estimates of diversity varied widely, with a nine-fold difference of observed species (17 to 158) recorded across pipelines. MetaPhlAn2 had the best diversity estimate, observing 17 species in comparison to the 19 within the reagent. Despite having significantly differing profiles for sensitivity and FPRA, Kaiju and MetaPhlAn2 both had the highest levels of similarity to the actual composition of the Gut-Mix-RR at ~ 75% for both bioinformatics pipelines.

We next evaluated the performance of the same bioinformatics tools using the Gut-HiLo reagent which challenges the ability to detect low abundance strains (Fig. 1). Broadly, the performance of each tool was similar to that for Gut-Mix, with MetaPhlAn2 the only tool with 0% FPRA and Kaiju the only tool to detect all species present in the reagent. For Centrifuge and MetaPhlAn2, sensitivity dropped when tested on Gut-Mix relative to Gut-HiLo, with MetaPhlAn2 dropping from 89 to 74% owing to a failure to detect the lowest abundant species. As with Gut-Mix, both Kaiju and MetaPhlAn2 remained the two tools with the highest measure of similarity in composition to the Gut-HiLo reagent, though Kaiju had a markedly higher measure of similarity than MetaPhlAn2.

**Fig. 2:**
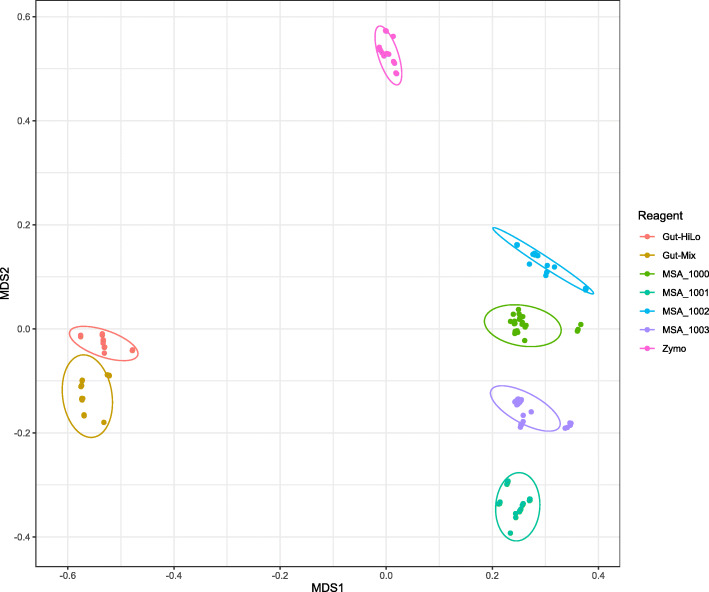
Visualisation of the relationship between different mock communities following sequencing and taxonomic profiling by a variety of approaches. A nMDS plot of a Bray-Curtis dissimilarity matrix was constructed from the species composition of five reference reagents following shotgun sequencing and taxonomic profiling by five different bioinformatics tools, MetaPhlAn2, Kaiju, Kraken, Bracken, and Centrifuge. Gut-HiLo = NIBSC Gut-HiLo-RR. Gut-Mix = NIBSC Gut-Mix-RR. MSA_1000 = ATCC MSA-1000. MSA_1001 = ATCC MSA-1001. MSA_1002 = ATCC MSA-1002. MSA_1003 = ATCC MSA-1003. Zymo = ZymoBIOMICS Microbial Community Standard

To investigate whether differences across bioinformatics tools could lead to differing conclusions on the relationships between microbial communities, we repeated the same analysis performed on the NIBSC reagents for the five commercially available mock communities with defined microbial communities (Fig. 2). Visualisation of the similarity between the microbial communities demonstrated that samples grouped were based on the reference reagent which was sequenced as opposed to the bioinformatics tool, the analysis was derived from (Fig. 2). Modelling the variance across community compositions demonstrated that reference reagent, from which sequences were generated, described the most variation in the data (Adonis, *R*^2^ = 0.91633 *F* = 0.000999), with bioinformatics tools used explaining approximately 5.29% of the variation in the dataset (Adonis, *R*^2^ = 0.05278, *F* = 0.000999). This suggests that although the choice of bioinformatics tool significantly influences species composition, it does not appear to change the underlying relationship between microbial communities. Combined with the results from the evaluation of pipeline performance using the four-measure reporting system, our data suggests that the choice of bioinformatics tool primarily influences measures of alpha diversity. This supports the rationale for having a multi-measure reporting system for reagents, as it allows for an accurate evaluation of the different aspects of results which most microbiome studies report on.

### Investigating the impact of strain composition on pipeline performance

**Fig. 3:**
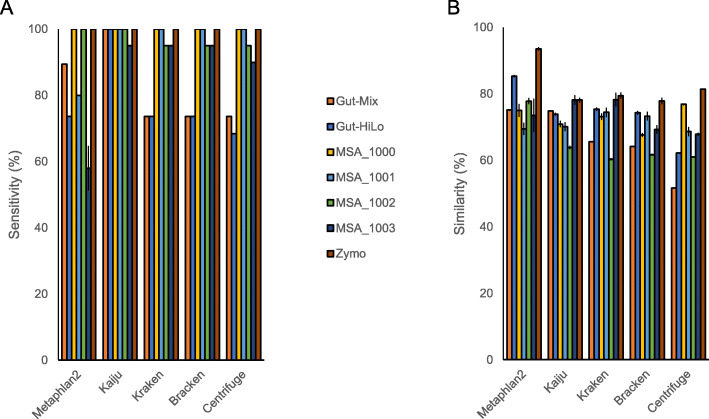
Changes in pipeline performance for sensitivity (**a**) and similarity (**b**) when using different reference reagents to benchmark bioinformatics tool performance. Gut-HiLo = NIBSC Gut-HiLo-RR. Gut-Mix = NIBSC Gut-Mix-RR. MSA_1000 = ATCC MSA-1000. MSA_1001 = ATCC MSA-1001. MSA_1002 = ATCC MSA-1002. MSA_1003 = ATCC MSA-1003. Zymo = ZymoBIOMICS Microbial Community Standard

There are a limited number of commercial reagents available for microbiome standardisation, although these are not considered accredited reference reagents. Indeed, there is no literature on what makes a suitable microbiome reference reagent, how different reference reagents might influence benchmarking studies, or how different microbial compositions may influence the accuracy of different pipelines. To understand how these factors may influence standardisation of the microbiome field, we investigated how five commercially available reference reagents compare in their ability to effectively benchmark bioinformatics tools. Using the sequences of these commercial mock communities, we calculated measures of sensitivity, FPRA, and similarity for five bioinformatics tools. These were then compared to the measures generated for the two NIBSC reference reagents. Across all tools, sensitivity and FPRA were significantly impacted depending on reference reagent choice, illustrating the variable nature of taxonomic profiling tools when changing microbial composition of the target sample (Fig. 3, Supplementary Table 3). For sensitivity, clear trends in tool performance were evident with tool performance was influenced by the number of strains present in a reagent and the composition which these strains were present (Fig. 3, Supplementary Tables 4 and 5). Across all tools, there was a lower sensitivity for reagents with a higher strain number and reagents with staggered concentrations of strains as opposed to even concentrations (Fig. 3, Supplementary Table 5). However, even considering composition and strain number, bioinformatics tools had a lower sensitivity for four of the five pipelines for NIBSC reagents in comparison to other reagents over a similar strain number and composition. This suggests that the species present also influences sequencing and bioinformatics tool performance, perhaps due to a GC content bias or their presence/absence in public databases [21, 22]. Similarity also significantly differed across reference reagents for different pipelines; however, changes were generally pipeline-specific, with no clear patterns emerging. Collectively, results demonstrate that different reference reagents will give different results for different pipelines. Hence, for effective bioinformatics tool benchmarking, purpose-specific reagents will be needed, which simulate the likely species composition of the target sample being analysed.

### Changing taxonomic resolution impacts pipeline performance

Strain level resolution is a desirable characteristic from bioinformatics tools for users investigating strain-level heterogeneity of detailed strain tracking [23]. In order to test the ability of bioinformatics tools to resolve a taxonomy lower than the species level, NIBSC-RRs included two subspecies of *Bifidobacterium longum*: *B. longum ssp. longum* and *B. longum ssp. Infantis*. At the sequenced depth, no pipeline could accurately resolve these two different subspecies, suggesting that strain or subspecies resolution is not possible using shallow shotgun metagenomics with the bioinformatics tools tested.

Frequently, microbiome studies report at different taxonomic levels depending on the hypothesis of the study and sequencing approaches used. We investigated how changing taxonomic levels may influence bioinformatics tool performance using the NIBSC-RRs. Using the four-measure reporting system to test tools at the genera level, tools significantly improved in performance for each reporting measure when using Gut-Mix-RR (Fig. 1, Supplementary Table 6). Four of the five tools had 100% sensitivity at the genera level and four of the five pipelines had < 1% FPRA. This had an impact on both diversity and similarity, with Kraken, Bracken, and Kaiju being the three tools with the highest similarity to the original composition. MetaPhlAn2 was the only bioinformatics tool to not significantly improve at the higher taxonomic classification but remained the tool with the best estimates of diversity of the Gut-Mix-RR (Fig. 1). For the Gut-HiLo-RR, classification level had less impact on measure performance, with only sensitivity being significantly improved by changing from species to genera level classification (Fig. 1, Supplementary Table 6). Across both reference reagents, improvements in tool performance when classifying at a higher taxonomic level were primarily due to reads previously assigned to incorrect species of the correct genera, now being classified as reads of the correct genera.

### Evaluation of 16S rRNA sequencing using NIBSC RRs

Amplicon sequencing of the16S rRNA gene is one of the most common methods for taxonomic profiling of the microbiome and is a proven effective tool for analysing broad-scale microbiome shifts in large cohort studies [24]. Considering its widespread use, we tested whether the NIBSC RRs and four-measure reporting system could effectively benchmark different 16S rRNA protocols and understand the biases they may introduce. Frequently, different primer sets are used in different microbiome studies, often amplifying different regions of the 16S rRNA gene [25]. Here, using the Gut-RRs, we tested the difference in performance between the V3-V4 and V4 region using two commonly cited primer sets S-D-Bact-0341-b-S-17/S-D-Bact-0785-a-A-21 targeting the V3-V4 region [26], and 515F(Parada)/806R(Apprill) targeting the V4 region [27, 28]. We also tested the performance of two of the most commonly used pipelines for analysing amplicon data, DADA2 [29] and Deblur [30], which were implemented through the QIIME2 platform [31].

Both primer sets were highly specific with < 0.01% FPRA; however, the 515F(Parada)/806R(Apprill) primers demonstrated significant improvements over the S-D-Bact-0341-b-S-17/S-D-Bact-0785-a-A-21 primer set in measures of sensitivity (94% vs 78%), similarity (80% vs 60%), and diversity estimates (16 observed genera vs 13 observed genera) for both tested bioinformatics pipelines (Fig. 1, Supplementary Table 7), supporting the widespread use of this primer set in global microbiome studies [24]. Comparison of DADA2 and Deblur pipelines for data generated by the 515F(Parada)/806R(Apprill) using both reference reagents suggested pipelines are comparable in performance. Both pipelines detected 15/16 present genera in both reagents with < 0.01% FPRA and both pipelines gave identical diversity estimates as measured by observed genera for the Gut-Mix-RR (15) and Gut-HiLo-RR (16). The only reporting measure to differ between pipelines was similarity, with taxonomic profiles from Deblur having a higher similarity to the actual compositions of Gut-Mix (84.47% vs 82.98%) and Gut-HiLo (77.12% vs 74.91%) than taxonomic profiles produced by DADA2 (Supplementary Table 8). An additional reporting measure which could be considered for amplicon sequencing is the number of recorded amplicon sequence variants (ASVs), a commonly used measure of sample diversity [32]. Deblur consistently estimated an ASV count of 24 across both reagents, an overestimation of 20% compared to the known number of strains. DADA2 estimated 26 ASVs for Gut-Mix-RR and 27 ASVS for Gut-HiLo-RR, an overestimation of 30 and 35% respectively. ASV estimates of biological diversity are likely inflated due to intragenomic variation in the 16S rRNA region [33]. Using publicly available genomes, we analysed the number of 16S rRNA genes per genome and the number of intragenomic variants. Across the 20 strains, there was a total of 53 possible 16S rRNA gene sequences. This highlights the problem of using ASVs as a measure of biological species diversity and supports results support previous findings relating to over-inflation of true strain diversity using DADA2 [34].

Rapid advancements in technology have resulted in multiple methods for the microbiome emerging in the last decade alone. Being able to assess accuracy across different methods is a crucial aspect of method development and of critical importance for ensuring comparability between studies. Using calculations of the four measure reporting system for both shotgun sequencing and 16S rRNA sequencing at the genera level, we investigated whether data from these two different sequencing strategies and associated bioinformatics tools could be accurately compared (Fig. 1, Supplementary Tables 9 – 14). Broadly the four-reporting results for 16S rRNA sequencing were similar to those obtained by shotgun sequencing. Differences were observed across all bioinformatics tools; however, these differences were tool/pipeline specific as opposed to being specifically biased by the library preparation used. For example, 16S rRNA sequencing pipelines were more sensitive than some shotgun sequencing tools (e.g. MetaPhlAn2) but less sensitive than others (e.g. Kaiju). Similarly, 16S rRNA sequencing pipelines had lower FPRA values than some shotgun tools (e.g. Centrifuge, Kaiju), but the same as others (MetaPhlAn2). This suggests that as previously reported, shallow shotgun sequencing and 16S rRNA technology results are comparable at the genera level [15], with the choice of bioinformatics pipeline being the key influence on the performance of the four reporting measures evaluated in this study.

## Discussion

Effective standardisation of methodologies used to analyse the microbiome is essential to the entire microbiome community [35]. Despite the microbiome field being established for over a decade, there are no accredited or certified reference materials available to the wider community. In this study, we describe the development of two NIBSC reference reagents for analysis of the microbiome, which can act as the first global working standards, and are candidate of WHO International Reference Reagents for NGS analysis of the microbiome. These reagents are part of a broader strategy for effective microbiome standardisation by NIBSC and will be followed by the development of whole-cell strain standards and spiked matrix standards to complement the DNA standards.

Standardisation of the microbiome field is complex as the methodologies used for study of the microbiome give semi-quantitative estimates of thousands of a priori unknown components. Hence, effective standardisation requires reporting measures which evaluate how accurately methods are capturing the abundance of thousands of a priori unknown microbes. The reporting system developed in this study combined with the two NIBSC DNA reference reagents allowed for a critical evaluation of the key biases of five commonly bioinformatics tools used to analyse shotgun sequencing through an Illumina platform. Reporting measures were designed to be general and simplistic to allow comparison between multiple methodology types. This ensures that reference reagents and the reporting framework are both compatible with older technologies and future-proof against newer technologies, allowing for traceability, continuity, and continuous innovation. We demonstrate here that reagents can be used with both 16S rRNA sequencing and shotgun sequencing on the Illumina platform. With novel technologies continually emerging, in the future these reference reagents will be tested with sequencing technologies other than those used here, such as long-read technologies and deep whole metagenomic sequencing.

DNA reference reagents allow for standardisation of library preparation, sequencing platform, and bioinformatics tools. There are several sequencing datasets available which are used to benchmark bioinformatics pipelines [22, 36]. Although good for initial tool development, they do not allow primary users to control for biases introduced during library preparation or sequencing. Different sequencing technologies will likely have their own specific biases, meaning that at laboratory level adoption of DNA reagents is required. Using DNA reference reagents will also allow users to capture the impact which sequence quality has on their final results. In this study, we exclusively used the Illumina MiSeq platform. Following quality control, we performed an independent evaluation of commonly used shotgun sequencing bioinformatics tools. The settings of each tool are adjustable but were left in this study at the settings recommended by the developers. This was deliberate to allow for a fair comparison across methodologies and to mimic how a typical user first approaches analysis. Under the recommended settings, the performance of different shotgun bioinformatics tools varied widely, supporting previous findings on the variability of shotgun sequencing pipelines [7, 37]. Differences in bioinformatics tools are likely due to both the settings, the databases which different tools use, and the fundamentally different algorithms underpinning the programs strategies for taxonomic profiling. For example, Kraken is based on an exact k-mer matching approach, MetaPhlAn2 is based on a unique clade-specific marker gene approach, and Kaiju translates nucleotides into amino acids and performed protein alignments. The variability across tools reported here supports previous studies which have demonstrated similar findings for other methodologies [5, 7, 22, 37, 38]. In this study, sequencing depth was consistent at the depth recommended for shallow shotgun sequencing [15]; however, it should be noted that tools could perform differently at different sequencing depths under the same settings.

For effective standardisation, global adoption of appropriate reference reagents is required. We demonstrate that the commercially available generic microbiome reference reagents are not appropriate for standardising the gut microbiome due to the inherent changes in pipeline performance, which occurs when changing reference reagents. A variety of factors ranging from GC content to presence of the sequence in databases can impact the successful sequencing and reporting of a strain in a community [21, 22]. Reagents which include strains which are not in the gut give an inaccurate picture on the likely taxonomy and GC content of the target sample. Benchmarking using these reagents, therefore, could give unrealistic estimates of pipeline performance particularly for reagents which are relatively simplistic. In the future, it is likely that purpose-specific reference reagents are needed, for each specific microbiome site measured (e.g. Skin, Lung, Oral, Vaginal).

Widespread adoption of the reference reagents and reporting system developed in this study could be an effective way for users to understand the biases they are introducing into their gut microbiome studies. In particular, it is concerning to see key measures of gut health such as ‘diversity’, being overestimated by as high as eight-fold for using some pipelines. To harmonise results across studies, in the future we will aim to establish key thresholds for each of these reporting measures to set benchmarks for what pipelines should have to achieve for data to be published and be accepted by the microbiome community. To do this, we will be proposing a collaborative study with multiple expert laboratories to take place during 2020, which should establish a consensus across the wider community. The study will also serve to have the reference reagents evaluated and submitted for endorsement by WHO as the first WHO International Reference Reagents for NGS analysis of the microbiome.

## Conclusions

We have developed two reference reagents and a reporting system which can help standardise the microbiome field. Testing of these reference reagents demonstrates that they can accurately evaluate differences in bioinformatics pipelines and reveal the staggering variability across a range of shotgun sequencing taxonomic profilers. Agreeing consensus thresholds for what users should achieve when using such reagents could prevent incorrect reporting of data and allow harmonisation of the field. Importantly, reference reagents were fit for purpose when using both 16S rRNA sequencing and shotgun sequencing and their use allowed for comparability of the biases of the two different approaches. Due to the variability in pipeline performance observed following the use of generic reference reagents, it is highly likely that site or purpose-specific reference reagents will be required in the future to ensure that pipelines are correctly benchmarked.

## Methods

### Strain selection

Prior studies such as the Human Microbiome Project and MetaHIT, have given a comprehensive catalogue of strains observed in the gut [2, 3]. As the microbiome varies on an individual basis and including all known strains to occur in the gut would not be practical, we focussed including genera which are known to have multiple abundant species and strains present in the healthy human gut. This allowed us to develop a standard which could test pipelines ability to distinguish between genera in the same family, species in the same genera, and strains in the same species. In total, we developed a 20-strain reference reagent (Table 1) comprising strains obtained from Leibniz Institute DSMZ-German Collection of Microorganisms and Cell Cultures GmbH (DSMZ).

### Generation of reference reagents

Strains were cultured as recommended by the supplier DSMZ (Table 1). Strains were checked for purity using agar streak plating and phase-contrast microscopy. Once purity was confirmed, strains were cultivated as recommended by supplier DSMZ, with strains harvested for DNA extraction. DNA was extracted using the Qiagen DNeasy PowerSoil kit (Qiagen, Manchester, UK). This kit was chosen on the basis that it is frequently used to extract DNA from samples for microbiome studies, such as in the Earth Microbiome Project [24]. Integrity of DNA was checked using agarose gel electrophoresis, with DNA visually checked to ensure size between 5–> 20 kb. In brief, 0.8% agarose (Cambridge Reagents, Cambridge, UK) gels were ran for 1 h at 100 v prior to visualisation using (Syngene Pxi Gel-Doc System). Following size validation, DNA was quantified using qubit fluorometric quantification (Thermo Fisher Scientific, UK). Each strain was measured on five separate occasions with an average reading taken as the final concentration value. To ensure strains were not contaminated, DNAs were then subjected to 16S rRNA PCR using the universal 27F and 1492R primers using Platinum Taq DNA Polymerase (Thermo Fisher Scientific, UK). PCR products were purified using a Qiagen PCR purification kit (Qiagen, Manchester, UK) and sequencing at Source Biosciences, Cambridge. Sequences were BLAST against the NCBI nr/nt database for strain identification. Following correct identification, DNA libraries for shotgun sequencing were constructed using the Nextera XT Kit (Illumina, USA) and sequenced paired end with 150 bp read length on a NextSeq 500 platform (Illumina, USA) using a NextSeq 500 Reagent Kit v2.5 (Illumina, USA). Resulting sequences were analysed using MetaPhlAn2 [16] to ensure no contamination was present. Following the cultivation and validation of all 20 strains, DNA was mixed to a final concentration of 10 ng/μl for the Gut-Mix-RR with all strains added to a concentration of 10 ng/μl. For Gut-HiLo, the final concentration of the reagent was 7.85 ng/μl, with strains added at a higher concentration of 20 ng/μl, a middle concentration of 2 ng/μl, and a low concentration of 0.2 ng/μl (Table 1). Integrity of the final reagents (pooled DNAs) was further assessed using Aglient TapeStation 2200 gDNA assay following manufacturer’s instructions. Briefly 1 μl of extracted DNA samples were added to 10 μl of gDNA sample buffer and run on a gDNA ScreenTape. TapeStation plots DNA fragment size (bp) against sample intensity (FU) generating a DNA Integrity Number (DIN) that determines the level of sample degradation. TapeStation allows for an accurate assessment of DNA quality and quantity. In all cases, reagents were over a DNA integrity score of 7.0 indicating highly intact genomic DNA. Evaluation of concentration by TapeStation showed good concordance range of the results from qubit fluorimetry (within a 15% range), giving additional assurance on estimation of DNA concentration. For final relative abundance, strains were adjusted for genome copy size (Table 1); hence, relative abundance represents the relative proportion in numbers of genomes of each strain. Genome sizes and percentage GC content were taken from The Bacterial Diversity Metadatabase (https://bacdive.dsmz.de/ Last Accessed on the 21 May 2020) and IMG/M [21]. Qubit was chosen as the primary method for giving estimated concentrations based on genome copy number. This allows a high level of comparability and utility with NGS protocols, such as Illumina, which recommend quantifying libraries using a fluorometric quantification method that uses dsDNA binding dyes [39]. Pooled DNAs, in the form of NIBSC Gut-Mix-RR and NIBSC Gut-HiLo-RR, were further evaluated using next generation 16S rRNA sequencing and shotgun sequencing. Shotgun and 16S rRNA sequencing analysis of the pooled DNAs, although variable across tools, widely gave results which would be expected based on pooling of the validated constituents. As part of our ongoing QC, we perform real-time monitoring of heterogeneity and degradation of all materials. Our product labels state the most up-to-date information on stability and storage available at the time.

### Shotgun sequencing

The NIBSC Gut-Mix-RR Gut-HiLo-RRs, ATCC MSA-1000, MSA-1001, MSA-1002, and MSA-1003 reagents, and ZymoBIOMICS Microbial Community DNA Standard libraries for shotgun sequencing were constructed using the Nextera XT Kit (Illumina, USA) and sequenced paired end with 300 bp read length on a MiSeq platform (Illumina, USA) using a MiSeq Reagent Kit v3 (Illumina, USA). NIBSC reference reagents were sequenced on the same sequencing run, ATCC reagents across two sequencing runs, and ZymoBIOMICS on its own sequencing run.

### Taxonomic profiling of shotgun metagenomic data

A detailed methodology including commands executed for the analysis of shotgun sequencing files, tool versions, and database versions can be found in the Supplementary Methods. All sequencing files (fastq) are publicly available through the NCBI Sequence Read Archive (NCBI Bioproject ID PRJNA622674). In brief, following sequencing, FastQC (http://www.bioinformatics.babraham.ac.uk/projects/fastqc/) was used to make initial judgements on data quality. In particular, the ends of the second (R2) reads were of low quality, as has previously been observed for longer Illumina reads. Quality control was employed using BBDuk (https://sourceforge.net/projects/bbmap/) with quality trimming at *Q* = 25 and minimal length filtering of trimmed reads at 100 bp. Sequence files were subsampled to 250,000 reads for R1 and R2 reads using seqtk (https://github.com/lh3/seqtk), making a total of 500,000 reads, the depth previously described for shallow shotgun metagenomics [15]. Following QC, shotgun sequencing data was analysed using the programs, MetaPhlAn2 [16], Kaiju [19], Kraken [17], Bracken [18], and Centrifuge [20]. Programs were chosen based on prevalence in the literature and because they offered a diversity of approaches to taxonomic profiling of metagenomic data. We acknowledge that there is a wealth of other programs for profiling shotgun sequencing data; however, the scope of this study was to validate the reference reagents, and not provide an exhaustive comparison of all possible methodologies. Considering this, programs were only tested in line with the developer’s recommendations in their tool’s tutorials (i.e. default), to allow for a fair comparison across programs. This also likely represents the settings which users would initially use when running the programs for the first time. The programs Kraken (and therefore Bracken) and Kaiju accepted paired-end reads. For MetaPhlan2 and Centrifuge, reads were combined into a single file prior to being processed. MetaPhlan2 does accept paired-end reads; however, the tool does not make use of the paired-end information; hence, this function was not used. Outputs from these programs were used to generate species and genera abundance tables. We then performed an additional quality filtering step of removing all species and or/genera below 0.005%, due to a substantial tail of low abundant species for several tools. This filtering was performed manually in Excel. Filtering of low abundant reads has been described as an effective measure of improving diversity estimates for Illumina sequencing data [40].

### 16S rRNA analysis

A detailed methodology including commands executed for the analysis of 16S rRNA sequencing files can be found in the Supplementary Methods. In brief, for each reference reagent, two sets of 16S rRNA sequencing data were generated based on two different primer sets. Amplicons were generated with Platinum Taq DNA Polymerase using S-D-Bact-0341-b-S-17/S-D-Bact-0785-a-A-21 targeting the V3-V4 region [26], and 515F(Parada)/806R(Apprill) targeting the V4 region [27, 28]. Amplicons were generated, purified, and sequenced according to Illumina manufacturer’s recommendations (https://support.illumina.com/documents/documentation/chemistry_documentation/16s/16s-metagenomic-library-prep-guide-15044223-b.pdf). Sequenced data was analysed through the QIIME2 (version 2019.7) with Deblur or DADA2 used for sequence quality control [29–31]. In brief, for analysis with DADA2, data was imported with the q2-cutadapt plugin used to remove primers and adapters. Reads without identifiable primer sequences were discarded. The q2-DADA2 plugin was then used to quality control sequence, merge error-corrected reads, and perform chimera/bimera removal. The q2-feature-classifier (sklearn) was used to assign taxonomy to representative sequences against the Silva database (132 release) [41]. Sequences were further filtered using the q2-feature-table plugin to ensure that only features which were present in all replicates of each respective reference reagent were included. Furthermore, as with shotgun data, all features which were less than 0.005% abundant for each replicate were removed. The q2-taxa plugin was used to generate taxa-bar plots which were used to extract relative genera abundances within each sample. For analysis with Deblur, following removal of primers and adapters with q2-cutadapt plugin, paired ends were joined using the q2-vsearch plugin. Sequences were then quality controlled using the q2-quality-filter plugin followed by the q2-deblur plugin. Following this, analysis was the same as for DADA2, with reads classified, filtered, and relative abundances of genera calculated.

The theoretical number of 16S rRNA sequence variants was calculated by using publicly available genomes (Table 1) which were analysed through IMG/M through the Sequence Alignment webtool [42].

### Calculation of the four reporting measures and statistical analyses

Using the species and genera abundance tables generated through bioinformatics analysis of the 16S rRNA sequencing files and shotgun sequencing files, we calculated measures of sensitivity, FPRA, diversity, and similarity based on mean average species or genera abundances of the five biological replicates. Measures of sensitivity, FPRA, diversity, and similarity were calculated using the following equations:

$$ \mathrm{Sensitivity}=\frac{\mathrm{Number}\ \mathrm{of}\ \mathrm{correctly}\ \mathrm{identified}\ \mathrm{species}}{\mathrm{Total}\ \mathrm{number}\ \mathrm{of}\ \mathrm{species}\ \mathrm{in}\ \mathrm{reagent}} \times 100 $$

$$ \mathrm{False}\ \mathrm{positive}\ \mathrm{relative}\ \mathrm{abundance}=\frac{\mathrm{Abundance}\ \mathrm{of}\ \mathrm{all}\ \mathrm{false}\ \mathrm{positive}\ \mathrm{species}}{\mathrm{Total}\ \mathrm{abundance}\ \mathrm{of}\ \mathrm{all}\ \mathrm{species}}\times 100 $$

$$ \mathrm{Diversity}=\mathrm{Total}\ \mathrm{number}\ \mathrm{of}\ \mathrm{all}\ \mathrm{observed}\ \mathrm{species}=\mathrm{Number}\ \mathrm{of}\ \mathrm{true}\ \mathrm{positive}\ \mathrm{species}+\mathrm{number}\ \mathrm{of}\ \mathrm{false}\ \mathrm{positive}\ \mathrm{species} $$

$$ {\mathrm{Similarity}}_{\left[ jk\right]}=1-\frac{\mathrm{sum}\mathrm{abs}\left(x\left[ ij\right]-x\left[ ik\right]\right)\ }{\mathrm{sum}\left(x\left[ ij\right]+x\left[ ik\right]\right)} $$

Where *x*[*ij*] and *x*[*ik*] refer to the quantity on species [*i*] in the actual species composition [*j*] and observed species composition [*k*] of the reagent. The vegdist function of the *R* vegan package can be used to calculate Bray-Curtis dissimilarity and was used in the current study, with 1—dissimilarity being used to calculate similarity*.*

The Kruskal-Wallis test was used to test for significance in changes for each reporting measure across bioinformatics tools and was calculated through *R* using the kruskal.test function [43]. The Wilcoxon signed-rank test was used to test for significance in improvements in tool performance when classifying at a species level against at a genera level. This was calculated in *R* using the wilcox.test function. The Dunn test was used as a post hoc test to the Kruskal-Wallis to evaluate significance in differences in reporting measures for each tool when compared with one another. This was done in *R* through the dunn.test function, To visualise similarity between communities analysed with each bioinformatics tool, a non-metric multidimensional scaling (nMDS) analysis using the Bray-Curtis dissimilarity was performed through the metaMDS function in the *R* vegan package and visualised using the ggplot function in the *R* ggplot2 package. A multivariate analysis of variance was performed using the adonis function the in *R* vegan package.

## Supplementary information

**Additional file 1.** Supplementary Methods.

**Additional file 2: Supplementary Table 1.** – Standard deviation of mean averages of biological replicates for measures of Sensitivity, FPRA, Diversity, and Similarity for all bioinformatic tools for all tested reagents. **Supplementary Table 2.** - Changes in bioinformatic pipeline performance evaluated using the NIBSC Gut-Mix-RR and Gut-HiLo-RR. **Supplementary Table 3.** – Changes in bioinformatic pipeline performance depending on reference reagent being sequenced. **Supplementary Table 4.** Changes in Sensitivity across all tested reagent as calculated by Dunn test with p values corrected by the Bonferroni Method. **Supplementary Table 5.** Changes in Sensitivity of bioinformatic pipelines associated with strain number and strain composition of the reference reagent being sequenced. **Supplementary Table 6.** Changes in measures associated with taxonomic level. **Supplementary Table 7.** Differences between the S-D-Bact-0341-b-S-17/S-D-Bact-0785-a-A-21 primer set and 515F(Parada)/806R(Apprill) primer set across both reagents. **Supplementary Table 8.** Differences in similarity between DADA2 and DEBLUR. **Supplementary Table 9.** Changes in Sensitivity for different taxonomic profiling pipelines for the Gut-Mix-RR as calculated by Dunn test with p values corrected by the Bonferroni Method. **Supplementary Table 10.** Changes in FPRA for different taxonomic profiling pipelines for the Gut-Mix-RR as calculated by Dunn test with p values corrected by the Bonferroni Method. **Supplementary Table 11.** Changes in Similarity for different taxonomic profiling pipelines for the Gut-Mix-RR as calculated by Dunn test with p values corrected by the Bonferroni Method. **Supplementary Table 12.** Changes in Sensitivity for different taxonomic profiling pipelines for the Gut-HiLo-RR as calculated by Dunn test with p values corrected by the Bonferroni Method. **Supplementary Table 13.** Changes in FPRA for different taxonomic profiling pipelines for the Gut-HiLo-RR as calculated by Dunn test with p values corrected by the Bonferroni Method. **Supplementary Table 14.** Changes in Similarity for different taxonomic profiling pipelines for the Gut-HiLo-RR as calculated by Dunn test with p values corrected by the Bonferroni Method.

## Data Availability

All sequencing data generated through this study is publicly available through the NCBI Sequence Read Archive upon publication (NCBI Bioproject ID PRJNA622674). All NIBSC generated reference reagents are available through request from the corresponding author. From 2020 onwards they will be made available through the NIBSC website. https://www.nibsc.org/

## Abbreviations

ASV: Amplicon sequence variant
FPRA: False positive relative abundance
NGS: Next-generation sequencing
NIBSC: National Institute for Biological Standards and Control
QC: Quality control
WHO: World Health Organization
