# Supplementary material for: Developing standards for the microbiome field
Source: Microbiome. 2020 Jun 26;8:98. doi: 10.1186/s40168-020-00856-3 (PMC7320585; doi:10.1186/s40168-020-00856-3)
Supplement: Supplementary file 2 — Additional file 1. Supplementary Methods. [file 40168_2020_856_MOESM1_ESM.docx]

**Supplementary Methods**

**Analysis of Shotgun Sequencing**

For analysis of shotgun metagenomic sequencing data we used the tools:

BBDuk <https://sourceforge.net/projects/bbmap/> version 38.73 [last accessed 22/11/2019]

seqtk <https://github.com/lh3/seqtk> [last accessed 22/11/2019]

Metaphlan2 <https://bitbucket.org/biobakery/metaphlan2/src/default/> [last accessed 22/11/2019]

Kaiju <http://kaiju.binf.ku.dk/> version 1.7.2. [last accessed 22/11/2019]

Kraken <https://github.com/DerrickWood/kraken> [last accessed 22/11/2019]

Bracken <https://ccb.jhu.edu/software/bracken/> v.2.0.0 [last accessed 22/11/2019]

All databases used were ones recommended by the developers in their tutorials or README files including the *mpa_v20_m200* database for Metaphlan2, the *NCBI RefSeq database* [last accessed 22/11/2019]for Kaiju, *Bacteria, Archaea (compressed)* for Centrifuge [last updated 4/15/2018], and the ‘Standard Kraken Database’ for Kraken and Bracken [last accessed 22/11/2019].

Sequencing files were generated using the Illumina MiSeq platform as detailed in the 'Methods' section of the main manuscript. All raw input and subsampled files are available through the Sequence Research Archive (NCBI Bioproject ID PRJNA622674).

In the following list of commands, ‘forward.fastq’ and ‘reverse.fastq’ denote the forward and reverse fastq files generated from a single replicate of a single sample (reference reagent). All replicates of all samples were analysed using the same approach and same settings.

1) Files forward.fastq and reverse.fastq were in the directory 'standardsanalysis'. We set this as our current working directory.

standardsanalysis=/Users/Greg/Documents/StandardsAnalysis

cd $standardsanalysis

2) We used the tool BBDuk to quality control the sequences. The GUI tool FASTQC was used to manually evaluate the quality of sequencing files from which decisions were made on settings for BBDuk. An array of settings were tested ranging from changing qtrim scores from 10 to 30 and testing no forced trimming and substantial forced trimming. Below are the final settings which were due to giving high quality scores across the length of the read and removing adapters. The same settings were used for every file to ensure no biases were introduced at this stage.

bbduk.sh -Xmx20g in1=forward.fastq in2=reverse.fastq out1=cleanforward.fastq \

out2=cleanreverse.fastq ref=/Users/Greg/bbmap/resources/adapters.fa ktrim=r k=21 mink=10 \

hdist=2 tpe tbo qtrim=r trimq=25 ftl=15 ftr=225 minlen=100

3) To subsample files, we used the tool seqtk. Reads were subsampled to 250,000 per file and combined to give 500,000 reads total. Combined files were used with Metaphlan2 and Centrifuge. Paired end files were used with Kraken, Bracken and Kaiju.

seqtk sample -s100 cleanforward.fastq 250000 > cleanforward_250k.fastq

seqtk sample -s100 cleanreverse.fastq 250000 > cleanreverse_250k.fastq

cat cleanforward_250k.fastq cleanreverse_250k.fastq > cleancombined_500k.fastq

4) We performed Metaphlan2 analysis of the combined sequencing files using the --ignore_viruses flag to prevent complications in calculations arising from classification of prophage.

mkdir Metaphlan2

metaphlan2.py $standardsanalysis/cleancombined_500k.fastq --input_type fastq \

--ignore_viruses --nproc 8 > $standardsanalysis/Metaphlan2/profile.txt

merge_metaphlan_tables.py \

$standardsanalysis/Metaphlan2/profile.txt > $standardsanalysis/Metaphlan2/merged_table.txt

5) To perform analysis using Kaiju we used paired end reads sequencing files with a filter (-c 12) to remove any reads which were under 0.005% abundance.

mkdir Kaiju

kaiju -t user/Greg/kaiju/kaijudb/nodes.dmp \

-f user/Greg/kaiju/kaijudb/kaiju_db.fmi \

-i $standardsanalysis/cleanforward_250k.fastq \

-j $standardsanalysis/cleanreverse_250k.fastq \

-o $standardsanalysis/Kaiju/output.txt -z 7 -a mem

kaiju2table -t user/Greg/kaiju/kaijudb/nodes.dmp -n user/Greg/kaiju/kaijudb/names.dmp \

-r species -c 12 -u -o $standardsanalysis/Kaiju/species.txt $standardsanalysis/Kaiju/output.txt

kaiju2table -t user/Greg/kaiju/kaijudb/nodes.dmp -n user/Greg/kaiju/kaijudb /names.dmp \

-r genus -c 12 -u -o $standardsanalysis/Kaiju/genus.txt $standardsanalysis/Kaiju/output.txt

6) To perform analysis using Centrifuge we used combined sequencing files.

mkdir Centrifuge

centrifuge -x /user/centrifuge/indices/p_compressed \

-U $standardsanalysis/cleancombined_500k.fastq -p 7 -S $standardsanalysis/Centrifuge/output \

--report-file $standardsanalysis/Centrifuge/report.txt

7) To perform analysis using Kraken and Bracken we used paired end sequencing files. Kraken and Bracken were run on a different machine to other tools listed here. The directory /home/AD/gamos contained the output from step 2 which were the subsampled sequencing files ‘cleanforward_250k.fastq’ and the ‘cleanreverse_250k.fastq’.

forward=/home/AD/gamos/cleanforward_250k.fastq

reverse=/home/AD/gamos/cleanreverse_250k.fastq

outdir=/home/AD/gamos/kraken

mkdir -p $outdir

cd $outdir

/usr/local/bin/kraken --threads 4 --paired --preload --db /raid/kraken/standard_kraken_db/ \

--classified-out $outdir/kraken.classified --unclassified-out $outdir/kraken.unclassified \

--out $outdir/kraken/kraken.output $forward $reverse

/usr/local/bin/kraken-mpa-report --db /raid/kraken/standard_kraken_db/ \

$outdir/kraken.output > $outdir/kraken.mpa.report

kraken-report --db --db /raid/kraken/standard_kraken_db/ \

$outdir/kraken.output > $outdir/kraken.report

bracken -d /raid/kraken/standard_kraken_db/ \

-i $outdir/kraken.report -o $outdir/bracken.report -r 140 -t 5

The primary outputs from the bioinformatic tools which were; 'merged _table.txt' from Metaphlan2 which was used to create species and genus abundance tables in Excel for all samples; the 'species.txt' and 'genus.txt' files from Kaiju were used to create species and genus abundance tables in Excel for all samples; the 'report.txt' file from Centrifuge was used to create species and genus abundance tables in Excel for all samples; the 'kraken.report' and 'bracken.report' files from kraken and bracken respectively which were used to create species and genus abundance tables in Excel for all samples. Identified species and genera below 0.005 % abundance of those reads which were classified, were filtered post-analysis from the species abundance and genus abundance tables. This was done using Excel. Final relative abundances were calculated relative to those species or genera which were classified and over 0.005 % abundance. Species and genus profiles were used to calculate the four reporting measures as described in the Methods of the main manuscript.

**Analysis of 16S rRNA sequencing**

To analyse 16S rRNA sequencing data we used the QIIME2 platform (QIIME2 2019.7) <https://qiime2.org/>. We tested two different routes of analysis through QIIME2. One route used the tool DADA2 for denoising and merging paired end reads. The second route used VSEARCH for paired end merging and Deblur for denoising. Two different primer sets were used to generate amplicons tested in this study which were sequenced on an Illumina MiSEQ platform. Fastq files generated from the 515F(Parada)/806R(Apprill) primer set and fastq files generated from the S-D-Bact-0341-b-S-17/S-D-Bact-0785-a-A-21 primer set were in different directories and processed separately. Fastq files were then separated by reagent, with each reagent processed separately. All Fastq files are available through the Sequence Research Archive (NCBI Bioproject ID PRJNA622674).

For analysis of sequencing data generated from the 515F(Parada)/806R(Apprill) primer set we used the following commands. We only detail analysis of one reagent here, however each reagent was processed the same except for steps 7 and 14, where the abundance threshold was calculated for each reagent based on total number of reads in the corresponding feature table.

1) The directory for the Gut-Mix fastq files were set to the current working directory

V4GutMix=/Users/Greg/Documents/16SAnalysis/GutMix

cd $V4GutMix

2) We next ‘imported’ the data into QIIME2 creating a .qza QIIME2 artifact:

qiime tools import \

--type 'SampleData[PairedEndSequencesWithQuality]' \

--input-path $V4GutMix \

--input-format CasavaOneEightSingleLanePerSampleDirFmt \

--output-path $V4GutMix/demux.qza

3) Primers and adaptors were removed using the q2-cutadapt plugin:

qiime cutadapt trim-paired \

--i-demultiplexed-sequences $V4GutMix/demux.qza \

--p-cores 4 \

--p-adapter-f GTGYCAGCMGCCGCGGTAA...ATTAGAWACCCBNGTAGTCC \

--p-adapter-r GGACTACNVGGGTWTCTAAT...TTACCGCGGCKGCTGRCAC \

--output-dir $V4GutMix/trimmed

4) We merged and denoised the reads using the q2-dada2 plugin.

qiime dada2 denoise-paired \

--i-demultiplexed-seqs $V4GutMix/trimmed/trimmed_sequences.qza \

--p-trunc-len-f 240 \

--p-trunc-len-r 200 \

--p-n-threads 0 \

--output-dir $V4GutMix/DADA2

5) To classify features, we used the q2-feature-classifier plugin using the classify sklearn method. We used a pre-trained classifier which was trained on the Silva 132 release with 99% OTUs from the 515F/806R. This was sourced from QIIME2 resources https://docs.qiime2.org/2019.10/data-resources/ [last accessed 22/11/2019].

qiime feature-classifier classify-sklearn \

--i-classifier /Users/Greg/Documents/Silva/silva-132-99-515-806-nb-classifier.qza \

--i-reads $V4GutMix/DADA2/representative_sequences.qza \

--o-classification $V4GutMix/DADA2/taxonomy.qza \

--p-n-jobs -1

6) We filtered the feature table produced by DADA2 to ensure we only included features present in all replicates:

qiime feature-table filter-features \

--i-table $V4GutMix/DADA2/table.qza \

--p-min-samples 5 \

--o-filtered-table $V4GutMix/DADA2/sample-contingency-filtered-table.qza

7) To filter out all features which did not appear at an abundance of 0.005 % in the final feature table, we visualised the contingency filtered feature table to calculate 0.005 % of the total read abundance and used this as a --p-min-frequency in the q2-feature-table plugin (filter-features).

qiime feature-table summarize \

--i-table $V4GutMix/DADA2/sample-contingency-filtered-table.qza \

--o-visualization $V4GutMix/DADA2/sample-contingency-filtered-table.qzv

qiime tools view $V4GutMix/DADA2/sample-contingency-filtered-table.qzv

qiime feature-table filter-features \

--i-table $V4GutMix/DADA2/sample-contingency-filtered-table.qza \

--p-min-frequency 43 \

--o-filtered-table $V4GutMix/DADA2/final-filtered-table.qza

8) To generate taxa bar plots and visualize them we used the q2-taxa plugin:

qiime taxa barplot \

--i-table $V4GutMix/DADA2/final-filtered-table.qza \

--i-taxonomy $V4GutMix/DADA2/taxonomy.qza \

--o-visualization $V4GutMix/DADA2/V4GutMixbarplots.qzv \

--m-metadata-file $V4GutMix/metadata.txt

qiime tools view $V4GutMix/DADA2/V4GutMixbarplots.qzv

9) To analyse the data through the deblur route, we initially had to join the trimmed sequences using VSEARCH through the q2-vsearch plugin:

qiime vsearch join-pairs \

--i-demultiplexed-seqs $V4GutMix/trimmed/trimmed_sequences.qza \

--output-dir $V4GutMix/deblur

10) We next performed additional quality filtering using the q2-quality-filter plugin.

qiime quality-filter q-score-joined \

--i-demux $V4GutMix/deblur/joined_sequences.qza \

--o-filtered-sequences $V4GutMix/deblur/QC_demux_trimmed-joined.qza \

--o-filter-stats $V4GutMix/deblur/qc_demux_stats.qza

11) We then denoised the quality filtered files using the q2-deblur plugin.

qiime deblur denoise-16S \

--i-demultiplexed-seqs $V4GutMix/deblur/QC_demux_trimmed-joined.qza \

--p-trim-length 253 \

--o-representative-sequences $V4GutMix/deblur/rep-seqs-deblur.qza \

--o-table $V4GutMix/deblur/table-deblur.qza \

--p-jobs-to-start 4 \

--p-sample-stats \

--o-stats $V4GutMix/deblur/deblur-stats.qza

12) To classify features, we used the q2-feature-classifier plugin using the classify sklearn method. We used a pre-trained classifier which was trained on the Silva 132 release with 99% OTUs from the 515F/806R. This was sourced from QIIME2 resources https://docs.qiime2.org/2019.10/data-resources/ [last accessed 22/11/2019].

qiime feature-classifier classify-sklearn \

--i-classifier /Users/Greg/Documents/Silva/silva-132-99-515-806-nb-classifier.qza \

--i-reads $V4GutMix/deblur/rep-seqs-deblur.qza \

--o-classification $V4GutMix/deblur/taxonomy.qza \

--p-n-jobs -1

13) We filtered the feature table produced by deblur to ensure we only included features present in all replicates:

qiime feature-table filter-features \

--i-table $V4GutMix/deblur/table-deblur.qza \

--p-min-samples 5 \

--o-filtered-table $V4GutMix/deblur/sample-contingency-filtered-table.qza

14) To filter out all features which did not appear at an abundance of 0.005 % in the final feature table, we visualised the contingency filtered feature table to calculate 0.005 % of the total read abundance and used this as a --p-min-frequency in the q2-feature-table plugin (filter-features).

qiime feature-table summarize \

--i-table $V4GutMix/deblur/sample-contingency-filtered-table.qza \

--o-visualization $V4GutMix/deblur/sample-contingency-filtered-table.qzv

qiime tools view $V4GutMix/deblur/sample-contingency-filtered-table.qzv

qiime feature-table filter-features \

--i-table $V4GutMix/deblur/sample-contingency-filtered-table.qza \

--p-min-frequency 43 \

--o-filtered-table $V4GutMix/deblur/final-filtered-table.qza

15) To generate taxa bar plots and visualize them we used the q2-taxa plugin:

qiime taxa barplot \

--i-table $V4GutMix/deblur/final-filtered-table.qza \

--i-taxonomy $V4GutMix/deblur/taxonomy.qza \

--o-visualization $V4GutMix/deblur/V4GutMixbarplots.qzv \

--m-metadata-file $V4GutMix/metadata.txt

qiime tools view $V4GutMix/deblur/V4GutMixbarplots.qzv

Fastq files generated from the S-D-Bact-0341-b-S-17/S-D-Bact-0785-a-A-21 primer set were processed through steps 1 - 15 accounting for different folder names and file locations with the two exceptions that the cutadapt step was adapted for the S-D-Bact-0341-b-S-17/S-D-Bact-0785-a-A-21 primers and was input as follows:

qiime cutadapt trim-paired \

--i-demultiplexed-sequences $V3GutMix/demux.qza \

--p-cores 4 \

--p-adapter-f CCTACGGGNGGCWGCAG...GGATTAGATACCCBDGTAGTC \

--p-adapter-r GACTACHVGGGTATCTAATCC...CTGCWGCCNCCCGTAGG \

--output-dir $V3GutMix/trimmed

And the pre-trained classifier used in steps 5 and 12 was the Silva 132 release with 99% OTUs for full length sequences. This was sourced from QIIME2 resources https://docs.qiime2.org/2019.10/data-resources/ [last accessed 22/11/2019].

The CSV files from the barplots generated in steps 8 and 15 were used as genera abundance tables. Genera abundance profiles were used to calculate the four reporting measures as described in the Methods of the main manuscript.
