# Supplementary material for: Developing standards for the microbiome field
Source: Microbiome. 2020 Jun 26;8:98. doi: 10.1186/s40168-020-00856-3 (PMC7320585; doi:10.1186/s40168-020-00856-3)
Supplement: Supplementary file 3 — Additional file 2: Supplementary Table 1. – Standard deviation of mean averages of biological replicates for measures of Sensitivity, FPRA, Diversity, and Similarity for all bioinformatic tools for all tested reagents. Supplementary Table 2. - Changes in bioinformatic pipeline performance evaluated using the NIBSC Gut-Mix-RR and Gut-HiLo-RR. Supplementary Table 3. – Changes in bioinformatic pipeline performance depending on reference reagent being sequenced. Supplementary Table 4. Changes in Sensitivity across all tested reagent as calculated by Dunn test with p values corrected by the Bonferroni Method. Supplementary Table 5. Changes in Sensitivity of bioinformatic pipelines associated with strain number and strain composition of the reference reagent being sequenced. Supplementary Table 6. Changes in measures associated with taxonomic level. Supplementary Table 7. Differences between the S-D-Bact-0341-b-S-17/S-D-Bact-0785-a-A-21 primer set and 515F(Parada)/806R(Apprill) primer set across both reagents. Supplementary Table 8. Differences in similarity between DADA2 and DEBLUR. Supplementary Table 9. Changes in Sensitivity for different taxonomic profiling pipelines for the Gut-Mix-RR as calculated by Dunn test with p values corrected by the Bonferroni Method. Supplementary Table 10. Changes in FPRA for different taxonomic profiling pipelines for the Gut-Mix-RR as calculated by Dunn test with p values corrected by the Bonferroni Method. Supplementary Table 11. Changes in Similarity for different taxonomic profiling pipelines for the Gut-Mix-RR as calculated by Dunn test with p values corrected by the Bonferroni Method. Supplementary Table 12. Changes in Sensitivity for different taxonomic profiling pipelines for the Gut-HiLo-RR as calculated by Dunn test with p values corrected by the Bonferroni Method. Supplementary Table 13. Changes in FPRA for different taxonomic profiling pipelines for the Gut-HiLo-RR as calculated by Dunn test with p values corrected by the Bon [file 40168_2020_856_MOESM2_ESM.docx]

Supplementary Table 1 – Standard deviation of mean averages of biological replicates for measures of Sensitivity, FPRA, Diversity, and Similarity for all bioinformatic tools for all tested reagents

| **Metaphlan2** | **Sensitivity** | **FPRA** | **Diversity** | **Similarity** |
| --- | --- | --- | --- | --- |
| NIBSC-Gut-Mix | 0.00 | 0.03 | 0.00 | 0.18 |
| NISBC-Gut-HiLo | 0.00 | 0.19 | 0.00 | 0.09 |
| MSA 1000 | 0.00 | 0.49 | 0.00 | 0.33 |
| MSA 1001 | 0.00 | 0.24 | 0.00 | 1.37 |
| MSA 1002 | 0.00 | 0.12 | 0.00 | 0.25 |
| MSA 1003 | 0.00 | 1.26 | 0.00 | 0.44 |
| Zymo | 0.00 | 0.09 | 0.00 | 0.22 |
| **Kaiju** | **Sensitivity** | **FPRA** | **Diversity** | **Similarity** |
| NIBSC-Gut-Mix | 0.00 | 0.10 | 1.30 | 0.12 |
| NISBC-Gut-HiLo | 0.00 | 0.33 | 3.54 | 0.42 |
| MSA 1000 | 0.00 | 0.14 | 1.00 | 0.98 |
| MSA 1001 | 0.00 | 0.24 | 1.10 | 1.37 |
| MSA 1002 | 0.00 | 0.07 | 1.22 | 0.50 |
| MSA 1003 | 0.00 | 0.08 | 2.83 | 1.44 |
| Zymo | 0.00 | 0.11 | 1.67 | 0.78 |
| **Kraken** | **Sensitivity** | **FPRA** | **Diversity** | **Similarity** |
| NIBSC-Gut-Mix | 0.00 | 0.06 | 0.00 | 0.14 |
| NISBC-Gut-HiLo | 0.00 | 0.04 | 0.00 | 0.54 |
| MSA 1000 | 0.00 | 0.00 | 0.00 | 0.98 |
| MSA 1001 | 0.00 | 0.04 | 0.00 | 1.39 |
| MSA 1002 | 0.00 | 0.09 | 0.41 | 0.35 |
| MSA 1003 | 0.00 | 0.03 | 0.00 | 2.05 |
| Zymo | 0.00 | 0.01 | 0.00 | 0.97 |
| **Bracken** | **Sensitivity** | **FPRA** | **Diversity** | **Similarity** |
| NIBSC-Gut-Mix | 0.00 | 0.07 | 0.55 | 0.18 |
| NISBC-Gut-HiLo | 0.00 | 0.04 | 0.89 | 0.58 |
| MSA 1000 | 0.00 | 0.07 | 0.71 | 0.57 |
| MSA 1001 | 0.00 | 0.05 | 0.55 | 1.31 |
| MSA 1002 | 0.00 | 0.04 | 0.55 | 0.29 |
| MSA 1003 | 0.00 | 0.04 | 0.00 | 1.29 |
| Zymo | 0.00 | 0.01 | 0.00 | 0.96 |
| **Centrifuge** | **Sensitivity** | **FPRA** | **Diversity** | **Similarity** |
| NIBSC-Gut-Mix | 0.00 | 0.03 | 0.00 | 0.18 |
| NISBC-Gut-HiLo | 0.00 | 0.19 | 0.00 | 0.09 |
| MSA 1000 | 0.00 | 0.49 | 0.00 | 0.33 |
| MSA 1001 | 0.00 | 0.24 | 0.00 | 1.37 |
| MSA 1002 | 0.00 | 0.12 | 0.00 | 0.25 |
| MSA 1003 | 0.00 | 1.26 | 0.00 | 0.44 |
| Zymo | 0.00 | 0.09 | 0.00 | 0.22 |

Supplementary Table 2 - Changes in bioinformatic pipeline performance evaluated using the NIBSC Gut-Mix-RR and Gut-HiLo-RR

| Purpose of test | Test Used | Test Result |
| --- | --- | --- |
| Changes in Sensitivity between shotgun pipelines evaluated using Gut-Mix-RR | Kruskal-Wallis | chi-squared = 24, df = 4, p-value = 7.987e-05 |
| Changes in FPRA between shotgun pipelines evaluated using Gut-Mix-RR | Kruskal-Wallis | chi-squared = 22.407, df = 4, p-value = 0.0001663 |
| Changes in Diversity between shotgun pipelines evaluated using Gut-Mix-RR | Kruskal-Wallis | chi-squared = 23.678, df = 4, p-value = 9.267e-05 |
| Changes in Similarity between shotgun pipelines evaluated using Gut-Mix-RR | Kruskal-Wallis | chi-squared = 22.737, df = 4, p-value = 0.0001429 |
| Changes in Sensitivity between shotgun pipelines evaluated using Gut-HiLo-RR | Kruskal-Wallis | chi-squared = 24, df = 4, p-value = 7.987e-05 |
| Changes in FPRA between shotgun pipelines evaluated using Gut-HiLo-RR | Kruskal-Wallis | chi-squared = 23.256, df = 4, p-value = 0.0001126 |
| Changes in Diversity between shotgun pipelines evaluated using Gut-HiLo-RR | Kruskal-Wallis | chi-squared = 23.659, df = 4, p-value = 9.347e-05 |
| Changes in Similarity between shotgun pipelines evaluated using Gut-HiLo-RR | Kruskal-Wallis | chi-squared = 21.703, df = 4, p-value = 0.0002296 |

Supplementary Table 3 – Changes in bioinformatic pipeline performance depending on reference reagent being sequenced

| Purpose of test | Test Used | Test Result |
| --- | --- | --- |
| Changes in Sensitivity across shotgun pipelines for different reference reagents | Kruskal-Wallis | chi-squared = 101.12, df = 6, p-value < 2.2e-16 |
| Changes in Similarity across shotgun pipelines for different reference reagents | Kruskal-Wallis | chi-squared = 76.138, df = 6, p-value = 2.238e-14 |

Supplementary Table 4: Changes in Sensitivity across all tested reagent as calculated by Dunn test with p values corrected by the Bonferroni Method.

|  | Gut-HiLo | Gut-Mix | MSA_1000 | MSA_1001 | MSA_1002 | MSA_1003 |
| --- | --- | --- | --- | --- | --- | --- |
| Gut-Mix | -0.60419 |  |  |  |  |  |
|  | 0.8186 |  |  |  |  |  |
|  |  |  |  |  |  |  |
| MSA_1000 | -6.60833 | -6.00414 |  |  |  |  |
|  | 0.0000* | 0.0000* |  |  |  |  |
|  |  |  |  |  |  |  |
| MSA_1001 | -5.28667 | -4.68248 | 1.321666 |  |  |  |
|  | 0.0000* | 0.0000* | 0.5588 |  |  |  |
|  |  |  |  |  |  |  |
| MSA_1002 | -3.88948 | -3.28529 | 2.718857 | 1.39719 |  |  |
|  | 0.0006* | 0.0056* | 0.0295 | 0.5682 |  |  |
|  |  |  |  |  |  |  |
| MSA_1003 | -0.793 | -0.18881 | 5.815334 | 4.493667 | 3.096476 |  |
|  | 0.8556 | 0.8502 | 0.0000* | 0.0000* | 0.0098* |  |
|  |  |  |  |  |  |  |
| Zymo | -6.60833 | -6.00414 | 0 | -1.32167 | -2.71886 | -5.81533 |
|  | 0.0000* | 0.0000* | 0.5 | 0.4657 | 0.0262 | 0.0000* |

Supplementary Table 5: Changes in Sensitivity of bioinformatic pipelines associated with strain number and strain composition of the reference reagent being sequenced

| Purpose of test | Test Used | Test Result |
| --- | --- | --- |
| Shotgun pipelines change in Sensitivity depending on strain number in the tested reference reagent | Kruskal-Wallis | chi-squared = 93.037, df = 2, p-value < 2.2e-16 |
| Shotgun pipelines change in Sensitivity depending on the composition of the different strains | Kruskal-Wallis | chi-squared = 93.037, df = 2, p-value < 2.2e-16 |

Supplementary Table 6: Changes in measures associated with taxonomic level

| Purpose of test | Test Used | Test Result |
| --- | --- | --- |
| Shotgun pipelines, change in Sensitivity, species vs genera Gut-Mix | Wilcoxon Test | P = 0.0015 |
| Shotgun pipelines change in FPRA, species vs genera, Gut-Mix | Wilcoxon Test | P = 0.0045 |
| Shotgun pipelines change in Diversity, species vs genera, Gut-Mix | Wilcoxon Test | P = 8.1 x 10^-5^ |
| Shotgun pipelines change in Similarity, species vs genera, Gut-Mix | Wilcoxon Test | P = 0.0076 |
| Purpose of test, change in Sensitivity, species vs genera Gut-HiLo | Wilcoxon Test | P = 7.4 x 10^-5^ |
| Shotgun pipelines change in FPRA, species vs genera, Gut- HiLo | Wilcoxon Test | P = 0.096 |
| Shotgun pipelines change in Diversity, species vs genera, Gut- HiLo | Wilcoxon Test | P = 0.15 |
| Shotgun pipelines change in Similarity, species vs genera, Gut- HiLo | Wilcoxon Test | P = 0.099 |

Supplementary Table 7: Differences between the S-D-Bact-0341-b-S-17/S-D-Bact-0785-a-A-21 primer set and 515F(Parada)/806R(Apprill) primer set across both reagents

| Purpose of test | Test Used | Test Result |
| --- | --- | --- |
| Sensitivity Gut Mix | Wilcoxon Test | P = 2.5 x 10^-6^ |
| Similarity Gut Mix | Wilcoxon Test | P = 1.8 x 10^-6^ |
| Diversity Gut Mix | Wilcoxon Test | P = 0.00043 |

Supplementary Table 8: Differences in similarity between DADA2 and DEBLUR

| Reagent | Test Used | Test Result |
| --- | --- | --- |
| Gut-Mix-RR | Wilcoxon Test | P = 0.079 |
| Gut-HiLo-RR | Wilcoxon Test | P = 0.079 |

Supplementary Table 9: Changes in Sensitivity for different taxonomic profiling pipelines for the Gut-Mix-RR as calculated by Dunn test with p values corrected by the Bonferroni Method

|  | Bracken | Centrifuge | DADA2 | Deblur | Kaiju | Kraken |
| --- | --- | --- | --- | --- | --- | --- |
| Centrifuge | 0.00000 |  |  |  |  |  |
|  | 1.00000 |  |  |  |  |  |
|  |  |  |  |  |  |  |
| DADA2 | 2.60768 | 2.60768 |  |  |  |  |
|  | 0.0957 | 0.0957 |  |  |  |  |
|  |  |  |  |  |  |  |
| Deblur | 2.60768 | 2.60768 | 0.00000 |  |  |  |
|  | 0.0957 | 0.0957 | 1.00000 |  |  |  |
|  |  |  |  |  |  |  |
| Kaiju | 0.00000 | 0.00000 | -2.60768 | -2.60768 |  |  |
|  | 1.00000 | 1.00000 | 0.0957 | 0.0957 |  |  |
|  |  |  |  |  |  |  |
| Kraken | 0.00000 | 0.00000 | -2.60768 | -2.60768 | 0 |  |
|  | 1.00000 | 1.00000 | 0.0957 | 0.0957 | 1 |  |
|  |  |  |  |  |  |  |
| MetaPhlan2 | 3.911521 | 3.911521 | 1.30384 | 1.30384 | 3.911521 | 3.911521 |
|  | 0.0010* | 0.0010* | 1.00000 | 1.00000 | 0.0010* | 0.0010* |

Supplementary Table 10: Changes in FPRA for different taxonomic profiling pipelines for the Gut-Mix-RR as calculated by Dunn test with p values corrected by the Bonferroni Method

|  | Bracken | Centrifuge | DADA2 | Deblur | Kaiju | Kraken |
| --- | --- | --- | --- | --- | --- | --- |
| Centrifuge | 0.803676 |  |  |  |  |  |
|  | 1.00000 |  |  |  |  |  |
|  |  |  |  |  |  |  |
| DADA2 | -3.21471 | -4.01838 |  |  |  |  |
|  | 0.0137* | 0.0006* |  |  |  |  |
|  |  |  |  |  |  |  |
| Deblur | -3.21471 | -4.01838 | 0.00000 |  |  |  |
|  | 0.0137* | 0.0006* | 1.00000 |  |  |  |
|  |  |  |  |  |  |  |
| Kaiju | -1.093 | -1.89668 | 2.121707 | 2.121707 |  |  |
|  | 1.00000 | 0.6076 | 0.3556 | 0.3556 |  |  |
|  |  |  |  |  |  |  |
| Kraken | -1.31803 | -2.12171 | 1.896677 | 1.896677 | -0.22503 |  |
|  | 1.00000 | 0.3556 | 0.6076 | 0.6076 | 1.00000 |  |
|  |  |  |  |  |  |  |
| MetaPhlan2 | -3.21471 | -4.01838 | 0.00000 | 0.00000 | -2.12171 | -1.89668 |
|  | 0.0137* | 0.0006* | 1.00000 | 1.00000 | 0.3556 | 0.6076 |

Supplementary Table 11: Changes in Similarity for different taxonomic profiling pipelines for the Gut-Mix-RR as calculated by Dunn test with p values corrected by the Bonferroni Method

|  | Bracken | Centrifuge | DADA2 | Deblur | Kaiju | Kraken |
| --- | --- | --- | --- | --- | --- | --- |
| Centrifuge | 1.573894 |  |  |  |  |  |
|  | 1.00000 |  |  |  |  |  |
|  |  |  |  |  |  |  |
| DADA2 | -2.28369 | -3.85758 |  |  |  |  |
|  | 0.2351 | 0.0012* |  |  |  |  |
|  |  |  |  |  |  |  |
| Deblur | -3.05521 | -4.6291 | -0.77152 |  |  |  |
|  | 0.0236* | 0.0000* | 1.00000 |  |  |  |
|  |  |  |  |  |  |  |
| Kaiju | -1.51217 | -3.08607 | 0.771516 | 1.543033 |  |  |
|  | 1.00000 | 0.0213* | 1.00000 | 1.00000 |  |  |
|  |  |  |  |  |  |  |
| Kraken | -0.7098 | -2.28369 | 1.573894 | 2.34541 | 0.802377 |  |
|  | 1.00000 | 0.2351 | 1.00000 | 0.1996 | 1.00000 |  |
|  |  |  |  |  |  |  |
| MetaPhlan2 | 0.802377 | -0.77152 | 3.086066 | 3.857583 | 2.31455 | 1.512172 |
|  | 1.00000 | 1.00000 | 0.0213* | 0.0012* | 0.2167 | 1.00000 |

Supplementary Table 12: Changes in Sensitivity for different taxonomic profiling pipelines for the Gut-HiLo-RR as calculated by Dunn test with p values corrected by the Bonferroni Method

|  | Bracken | Centrifuge | DADA2 | Deblur | Kaiju | Kraken |
| --- | --- | --- | --- | --- | --- | --- |
| Centrifuge | 2.149317 |  |  |  |  |  |
|  | 0.3319 |  |  |  |  |  |
|  |  |  |  |  |  |  |
| DADA2 | 0.00000 | -2.14932 |  |  |  |  |
|  | 1.00000 | 0.3319 |  |  |  |  |
|  |  |  |  |  |  |  |
| Deblur | 0.00000 | -2.14932 | 0.00000 |  |  |  |
|  | 1.00000 | 0.3319 | 1.00000 |  |  |  |
|  |  |  |  |  |  |  |
| Kaiju | -2.14932 | -4.29863 | -2.14932 | -2.14932 |  |  |
|  | 0.3319 | 0.0002* | 0.3319 | 0.3319 |  |  |
|  |  |  |  |  |  |  |
| Kraken | 0.00000 | -2.14932 | 0.00000 | 0.00000 | 2.149317 |  |
|  | 1.00000 | 0.3319 | 1.00000 | 1.00000 | 0.3319 |  |
|  |  |  |  |  |  |  |
| MetaPhlan2 | 3.009044 | 0.859726 | 3.009044 | 3.009044 | 5.158361 | 3.009044 |
|  | 0.0275 | 1.00000 | 0.0275 | 0.0275 | 0.0000* | 0.02750 |

Supplementary Table 13: Changes in FPRA for different taxonomic profiling pipelines for the Gut-HiLo-RR as calculated by Dunn test with p values corrected by the Bonferroni Method

|  | Bracken | Centrifuge | DADA2 | Deblur | Kaiju | Kraken |
| --- | --- | --- | --- | --- | --- | --- |
| Centrifuge | 2.966782 |  |  |  |  |  |
|  | 0.0316 |  |  |  |  |  |
|  |  |  |  |  |  |  |
| DADA2 | 1.081639 | -1.88514 |  |  |  |  |
|  | 1 | 0.6238 |  |  |  |  |
|  |  |  |  |  |  |  |
| Deblur | -0.83441 | -3.80119 | -1.91605 |  |  |  |
|  | 1 | 0.0015* | 0.5813 |  |  |  |
|  |  |  |  |  |  |  |
| Kaiju | 2.194182 | -0.7726 | 1.112543 | 3.02859 |  |  |
|  | 0.2963 | 1.00000 | 1.00000 | 0.0258 |  |  |
|  |  |  |  |  |  |  |
| Kraken | 0.803503 | -2.16328 | -0.27814 | 1.637911 | -1.39068 |  |
|  | 1 | 0.3205 | 1.00000 | 1.00000 | 1.00000 |  |
|  |  |  |  |  |  |  |
| MetaPhlan2 | -1.66882 | -4.6356 | -2.75045 | -0.83441 | -3.863 | -2.47232 |
|  | 0.9991 | 0.0000* | 0.0625 | 1.00000 | 0.0012* | 0.141 |

Supplementary Table 14: Changes in Similarity for different taxonomic profiling pipelines for the Gut-HiLo-RR as calculated by Dunn test with p values corrected by the Bonferroni Method

|  | Bracken | Centrifuge | DADA2 | Deblur | Kaiju | Kraken |
| --- | --- | --- | --- | --- | --- | --- |
| Centrifuge | 2.746599 |  |  |  |  |  |
|  | 0.0632 |  |  |  |  |  |
|  |  |  |  |  |  |  |
| DADA2 | 1.203566 | -1.54303 |  |  |  |  |
|  | 1.00000 | 1.00000 |  |  |  |  |
|  |  |  |  |  |  |  |
| Deblur | -0.49377 | -3.24037 | -1.69734 |  |  |  |
|  | 1.00000 | 0.0125* | 0.9411 |  |  |  |
|  |  |  |  |  |  |  |
| Kaiju | -1.8825 | -4.6291 | -3.08607 | -1.38873 |  |  |
|  | 0.6276 | 0.0000* | 0.0213* | 1.00000 |  |  |
|  |  |  |  |  |  |  |
| Kraken | -0.52463 | -3.27123 | -1.7282 | -0.03086 | 1.357869 |  |
|  | 1.00000 | 0.0112* | 0.8815 | 1.00000 | 1.00000 |  |
|  |  |  |  |  |  |  |
| MetaPhlan2 | 1.975082 | -0.77152 | 0.771516 | 2.468853 | 3.857583 | 2.499714 |
|  | 0.5067 | 1.00000 | 1.00000 | 0.1423 | 0.0012* | 0.1305 |
